# Cloud‐Integrated Smart Nanomembrane Wearables for Remote Wireless Continuous Health Monitoring of Postpartum Women

**DOI:** 10.1002/advs.202307609

**Published:** 2024-01-26

**Authors:** Jared Matthews, Ira Soltis, Michelle Villegas‐Downs, Tara A. Peters, Anne M. Fink, Jihoon Kim, Lauren Zhou, Lissette Romero, Barbara L. McFarlin, Woon‐Hong Yeo

**Affiliations:** ^1^ IEN Center for Wearable Intelligent Systems and Healthcare at the Institute for Electronics and Nanotechnology Georgia Institute of Technology Atlanta GA 30332 USA; ^2^ George W. Woodruff School of Mechanical Engineering Georgia Institute of Technology Atlanta GA 30332 USA; ^3^ Department of Human Development Nursing Science College of Nursing University of Illinois Chicago 845 S. Damen Ave., MC 802 Chicago IL 60612 USA; ^4^ Department of Biobehavioral Nursing Science College of Nursing University of Illinois Chicago 845 S. Damen Ave., MC 802 Chicago IL 60612 USA; ^5^ Wallace H. Coulter Department of Biomedical Engineering Georgia Tech and Emory University School of Medicine Atlanta GA 30332 USA; ^6^ Parker H. Petit Institute for Bioengineering and Biosciences Institute for Materials Institute for Robotics and Intelligent Machines Georgia Institute of Technology Atlanta GA 30332 USA

**Keywords:** cloud integration, postpartum women, remote health monitoring, wearable electronics

## Abstract

Noncommunicable diseases (NCD), such as obesity, diabetes, and cardiovascular disease, are defining healthcare challenges of the 21st century. Medical infrastructure, which for decades sought to reduce the incidence and severity of communicable diseases, has proven insufficient in meeting the intensive, long‐term monitoring needs of many NCD disease patient groups. In addition, existing portable devices with rigid electronics are still limited in clinical use due to unreliable data, limited functionality, and lack of continuous measurement ability. Here, a wearable system for at‐home cardiovascular monitoring of postpartum women—a group with urgently unmet NCD needs in the United States—using a cloud‐integrated soft sternal device with conformal nanomembrane sensors is introduced. A supporting mobile application provides device data to a custom cloud architecture for real‐time waveform analytics, including medical device‐grade blood pressure prediction via deep learning, and shares the results with both patient and clinician to complete a robust and highly scalable remote monitoring ecosystem. Validated in a month‐long clinical study with 20 postpartum Black women, the system demonstrates its ability to remotely monitor existing disease progression, stratify patient risk, and augment clinical decision‐making by informing interventions for groups whose healthcare needs otherwise remain unmet in standard clinical practice.

## Graphical abstract

This paper reports a wearable system for at‐home cardiovascular monitoring of postpartum women – a group with urgently unmet NCD needs in the United States – using a cloud‐integrated soft sternal device with conformal nanomembrane sensors.

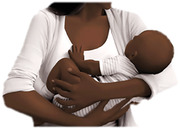

## 1 Introduction

Noncommunicable diseases (NCDs) have become entrenched as the primary sources of human mortality in the 21st century. The World Health Organization estimates that ≈3‐quarters of all deaths globally result from NCDs, such as diabetes, pulmonary disorders, cancer, and cardiovascular disease,^[^ ^1^ ^]^ with many proving chronic, debilitating, and ultimately fatal. Tragically, lifestyle characteristics play an important role in the genesis of these diseases,^[^ ^2^ ^]^ and they have become a persistent challenge to contemporary clinical infrastructures focused on acute treatment over prevention and long‐term monitoring. Clinical literature has long indicated that broadened screening, increased awareness, and improved clinical accessibility are crucial in mitigating the ongoing and worsening effects of these diseases.^[^ ^3^ ^]^ One manifestation of NCDs is postpartum mortality, a complication occurring after childbirth stemming frequently from preexisting cardiovascular disease (CVDs).^[^ ^4^ ^]^ Indeed, in conjunction with the increasing incidence of CVDs, pregnancy‐related mortality rates have increased in the United States for the past three decades, now ranking it last among peer nations for maternal outcomes and placing it among the few countries globally in which childbirth‐related mortality rates continue to increase in the 21st century.^[^ ^5^ ^]^ Underlying this tragedy are still higher rates of death among racial and ethnic minority groups, with Black women 2.6 times more likely to die due to pregnancy and giving birth compared to non‐Hispanic white women (69.9 deaths per 100 000 births compared to 26.6 deaths per 100 000 as of 2021).^[^ ^6^ ^]^ While the causes of this rising maternal mortality and its racial disparities are not fully understood, it is known that the postpartum period—the time beginning immediately after childbirth and lasting for up to 12 months after that—is a critical window for preventing maternal death; recent public health data show that 40% of all pregnancy‐related maternal deaths in the United States occur 1–42 days postpartum and over 50% of such deaths occur within the 1st year postpartum.^[^ ^4^, ^5^ ^]^ In line with trends for all women, the plurality of these deaths is attributed to CVD, including cardiomyopathy, sudden heart failure, and hypertensive disorders, the latter of which is the most common pregnancy‐related complication.^[^ ^7^ ^]^ With the current standard of postpartum care in the United States being a follow‐up 4–6 weeks after childbirth, many of these potentially lethal postpartum complications occurring in the 1st month are missed. Adding to this is the ineffectiveness of the follow‐up in addressing the needs of minority groups, with recent work finding that African–American and Hispanic women struggle to attend meetings due to deficits in transportation, access, and patient awareness.^[^ ^8^ ^]^ Thus, the women most vulnerable to NCD complications during the most vulnerable time after childbirth are the least likely to receive screening and any necessary interventional care.^[^ ^9^ ^]^

With recent advances in manufacturing,^[^ ^10^ ^]^ flexible electronics, and telemedicine, soft wearable sensors present compelling opportunities for addressing the challenges posed by NCDs in communities lacking sufficient clinical infrastructure. Wearables, noted for their cost‐effectiveness,^[^ ^11^ ^]^ are particularly well‐suited to postpartum monitoring given the close coupling of postpartum complications (and CVDs more broadly) with socioeconomic standing.^[^ ^12^ ^]^ But soft sensors need not sacrifice measurement fidelity for their accessibility; by reducing bulkiness and providing superior skin conformality compared to conventional rigid alternatives, they have had demonstrable success reducing motion artifacts and thereby improving signal‐to‐noise ratio for biosignal measurements.^[^ ^13^ ^]^ While capable of high‐quality sensing, the compact and flexible form factor of wearable devices also makes them amenable to long‐term, daily monitoring, which is critical given that both comfort and ease of use are deciding factors in the acceptance of wearables by patients across a spectrum of disease groups.^[^ ^14^ ^]^ Further, the proliferation of wireless protocols such as Bluetooth Low‐Energy (BLE) capable of supporting biosignal data throughputs, has made these devices compatible with major categories of consumer devices, including smartphones, tablets, and personal computers, permitting easy integration with technology already familiar to patients and already widely used in consumer health monitoring. However, only some reported wearable devices have shown compatibility with long‐term, at‐home use. Others need a greater breadth of sensors or derived metrics to characterize cardiovascular health, such as blood oxygen saturation or blood pressure. Further, most do not report being compatible with all major consumer mobile devices on the market, limiting their patient applicability. Notably, few have been validated through long‐term clinical studies, and none have seen use in postpartum contexts. Mirroring the lack of clinical solutions for increasingly severe postpartum mortality in the United States, there is a marked absence of prior work in the literature suitable for long‐term, at‐home use in postpartum monitoring.

Here, we introduce a soft, wearable, cloud‐interfaced device capable of recording high‐quality electrocardiography (ECG), heart rate (HR), heart rate variability (HRV), photoplethysmography (PPG)‐based blood oxygen saturation (SpO_2_), respiration rate (RR), skin temperature, blood pressure (BP) for postpartum cardiovascular monitoring. This work directly addresses the limitations in current clinical practice contributing to the increasingly severe maternal health crisis in the United States by enabling long‐term, remote, continuous, and cost‐effective monitoring of key cardiovascular health metrics. Postpartum women, who currently spend weeks outside the clinic during their most vulnerable time unattended, can continue to recover at home while providing themselves and their clinicians real‐time insight into their health, providing a feedback loop for clinical decision‐making where none currently exists. For this improvement over the status quo, we introduce innovations in the mechanical design of sternum‐based wearable sensors, provide real‐time delivery of key cardiovascular metrics via a multiplatform mobile application, and integrate deep learning for real‐time blood pressure prediction via the cloud. To our knowledge, as shown in **Table** **1**, no prior wearable systems validated in a clinical trial have achieved calibration‐free blood pressure prediction remotely. Indeed, our validation involved high‐ and low‐risk postpartum women in an at‐home month‐long study, wherein the device shared daily updates with clinical staff and detected key indicators of elevated postpartum risk, including tachycardia in the designated high‐risk group.

**Table 1:** Comparison of recently developed wearable cardiovascular monitoring systems.

Reference	Clinical validation		Telemedicine technology		Blood pressure prediction	
	Postpartum monitoring	Number of participants	Cloud integration	Multiplatform mobile app	Calibration‐free method	SBP/DBP error
This work	✓	20	✓	✓	✓	4.84 ± 4.16 2.86 ± 2.97
[16a]	−	−	✓	−	✓	Not specified
[16b]	−	−	✓	−	−	−
[30]	−	−	−	−	−	−0.16 ± 2.97 2.83 ± 1.68
[24d]	−	576	−	✓	−	0.6 0.2
[31]	−	−	✓	−	−	−
[32]	−	10	−	−	−	−
[33]	−	−	✓	−	−	−
[34]	−	−	−	−	−	−
[35]	−	−	−	−	−	−
[36]	−	−	−	−	−	−
[24e]	−	−	✓	−	−	−

SBP, systolic blood pressure; DBP, diastolic blood pressure

## 2 Results and Discussion

### 2.1 Design, Architecture, and Overview of a Soft Wearable System

**Figure 1:**
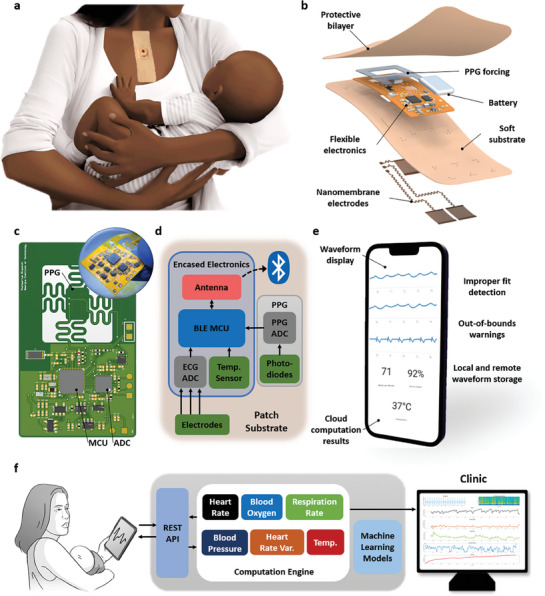
Overview of cloud‐integrated smart nanomembrane wearable for remote wireless continuous health monitoring of postpartum Black women. a) Illustrated postpartum clinical use case indicating device placement and form factor. b) The device's detailed structure shows a soft bilayer design, nanomembrane electrode, and flexible electronics. c) Illustrated device with an inset photo of a manufactured fPCB. d) Functional block diagram of the same circuit showing the device's sensors and Bluetooth‐enabled wireless communication unit. e) Patient‐facing multiplatform mobile application, showing real‐time waveforms and cloud computation. f) End‐to‐end view of the platform with cloud microservices expanded for at‐home use of the wearable health monitor.

In this study, we demonstrate real‐time, remote cardiovascular health monitoring of postpartum women using a cloud‐integrated mobile application, associated cloud architecture, and a wireless sternal patch. An illustration demonstrates a use case of the system for postpartum Black women, showing the anticipated sensing location of the device at the sternum (**Figure** **1**a). With amenability to daily monitoring an important design consideration for long‐term wearable device acceptability,^[^ ^14^ ^]^ the sternum was selected as a single sensing location, diverting from the multiple sensors liable to interfere with daily activities used for related applications in prior wearable systems. ECG and PPG, the key signals measured by the device, provide insight into cardiovascular health through the study of their waveform morphology and from the derivation of key metrics such as heart rate, blood oxygen saturation, and heart rate variability. A flexible passive forcing mechanism, illustrated along with the device's other key components in Figure 1b, was developed to improve PPG contact pressure at the sternum, which is known to enhance the quality of signal at this otherwise challenging PPG sensing location.^[^ ^13e^ ^]^ An encapsulating bilayer design projects this mechanism and other internal components from damage without compromising the flexibility or softness of the device. Within this bilayer, flexible electronics can provide ECG, PPG, and temperature sensing, as illustrated in Figure 1b,c and Figure S1 (Supporting Information). The fabrication process for the entire device is illustrated in Figure S2 (Supporting Information).

The wearable device implements the Bluetooth Low‐Energy (BLE) communication protocol for the wireless delivery of biosignal data to a tablet or smartphone with a custom mobile application, developed using Google's Flutter framework (Figure 1d,e). Flutter allows for multiplatform mobile development, meaning a single codebase can produce applications for both iOS and Android, simplifying the development, testing, and maintenance of the application. Further, targeting multiple operating systems ensures uniform compatibility with the estimated 90% of US consumers with a mobile device of any kind,^[^ ^15^ ^]^ a coverage that remains uncommon in wearable device systems. The mobile application serves multiple key functions in the system: it interfaces with the soft device to receive the raw waveform data, provides segments of these waveforms to a cloud‐based computation engine to process, saves the waveform data to the cloud for offline processing, and further saves this data to local device storage for future uploading if an internet connection cannot be established. The mobile application was carefully designed to impose a minimally constraining interface on prospective connections and reuse functionality between these connections, providing (though unused in the clinical study reported here) support for an ecosystem of devices by collecting and processing data from potentially multiple points of measurement simultaneously. The mobile application also provides access to the modern cloud technologies that underpin the system's remote monitoring ability. Modern cloud solutions are eminently scalable under load, rapidly deploying more servers to handle user requests in parallel. Despite being reliable and readily integrated with existing mobile software development kits, the cloud has received little attention in the wearable device field. Those prior works that include cloud technologies frequently leverage them in an internet‐of‐things capacity, with direct device connections to the cloud, typically via the Message Queuing Telemetry Transport protocol.^[^ ^16^ ^]^ While this simplifies the design process obtained by bypassing mobile device integration and application development, real‐time user feedback becomes difficult or impossible.

This work used the cloud in two primary ways: first, a cloud architecture shown in Figure 1e,f and Figure S1 (Supporting Information) provided users of the wearable device with real‐time characterization of their cardiovascular health through a low‐maintenance, highly scalable computation engine. The computation engine is *serverless* – that is, details of its resource provisioning, maintenance, and scaling under load are handled by the cloud provider, allowing greater research effort toward device development. The engine provides the computational power to process thousands of simultaneous waveform submissions to determine heart rate, respiration rate, blood pressure, heart rate variability, and blood oxygen saturation in real time. These are then returned to the client's mobile device for presentation. Cloud‐based signal quality was also determined by computing signal quality indices (SQIs). The application prompted users to correct the device placement given out‐of‐bounds values of these SQIs as described in,^[^ ^17^ ^]^ maintaining signal quality throughout each measurement session. Beyond real‐time calculations, the cloud secondarily provided a database for automatically uploading waveform files, enabling long‐term clinical monitoring and decision‐making through offline clinician access to patient data. An application programming interface was exposed to the patient's mobile device over the internet via an industry‐standard representational state transfer interface to parse, authenticate, and distribute requests to the engine instances such that no single instance is overwhelmed and latencies for the user remain low.

### 2.2 Study of Mechanics, Materials, and Optimal Contact Force

**Figure 2:**
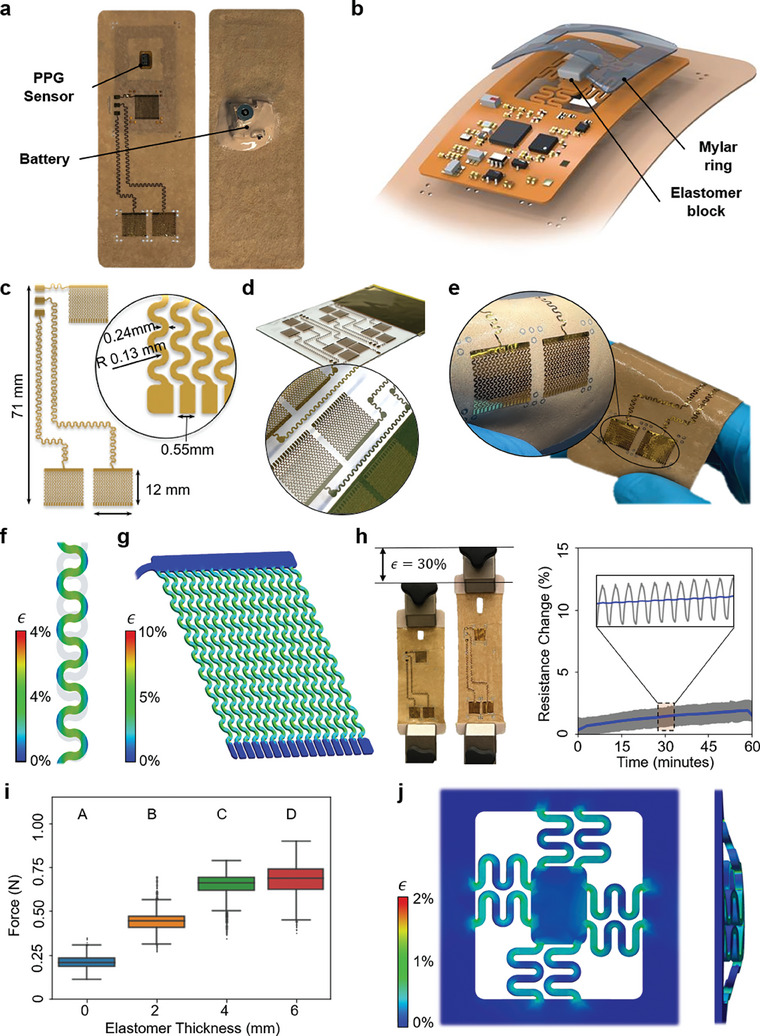
Soft device mechanical designs. a) Top‐down view of the device from both sides. b) PPG passive forcing mechanism to increase skin contact pressure. c) Nanomembrane electrode geometry, showing novel electrode pad design. d) View of laser‐micromachined electrodes. e) Integration of electrode pattern with adhesive underlayer. Both f) Maximum 4% local interconnect strain and g) maximum 10% local electrode strain for 10% overall device strain when constrained to unidirectional deformation; undeformed pattern in grey. h) Testing of electrode DC resistance (BK891 LCR meter) with 120 cycles to 30% device strain each over 1 h with 10 s moving mean shown in blue; maximum resistance change of 1.9% (0.4 Ω). i) PPG skin force distributions for various elastomer block thicknesses. j) True‐scale simulated strain of 1N uniform forcing on the PPG, with maximum strain in the serpentine pattern of 2%.

Robust mechanical characterization of the wearable device, shown in **Figure** **2**a, was necessary given its intended use in weeks‐long, unaided remote monitoring applications. Specifically, the nanomembrane electrode design and novel PPG forcing mechanism (Figure 2b) were evaluated in both simulation and laboratory testing. The electrodes were composed of copper, chromium, and gold layers of electron‐beam deposited on polyimide (PI) film to provide a skin‐safe, flexible form factor known to exceed the signal‐to‐noise ratio (SNR) typically obtained rigid clinical alternatives for ECG measurement. The sensing portion of the electrode (Figure 2c), while taking inspiration from prior conformal nanomembrane electrodes, permits long‐term, unaided application and removal without electrode delamination from the patch substrate. Building on the conventional “Greek cross” fractal serpentine pattern,^[^ ^18^ ^]^ pairs of opposite ends of each electrode were affixed to the fabric substrate with a thin layer of skin‐safe elastomer, eliminating delamination from normal forces. Avoiding this delamination was found in preliminary testing to be critical for long‐term monitoring because, once initiated, the delamination propagated readily down the length of the flexible electrode and rendered the device unable to sense ECG. Two such failure modes are shown in Figure S3 (Supporting Information). The redesign necessitated the use of a femtosecond pulse duration laser for extracting the pattern from the electron beam‐coated PI, as the density of electrode patterns seen immediately after cutting in Figure 2d and on the patch in Figure 2e was not achievable with conventional pulse durations that would otherwise thermally load the substrate. Finite element analysis was used to explore this new electrode pattern. Uniaxial electrode strains of 15% were applied to observe the maximum local strain in the serpentine designs, both within the interconnects and the electrode pads. Figure 2f,g show maximum local strains of 8% and 10% for the interconnect and sensing pattern, respectively, indicating a marginal reduction of strain with this design. Expanding on this, real‐world characterization of the electrode in Figure 2h shows mechanical and electrical robustness in response to large cyclic strain (30%). Electrode resistance is plotted for 100 cycles over the course of an hour, with average resistance increasing by 2%, within acceptable bounds for ECG collection. Prior work has indicated that measuring high‐quality PPG from the sternum is feasible for long periods given an applied force on the PPG sensor that overcomes the lack of local vascularization.^[^ ^13e^ ^]^ Indeed, early device iterations lacking deliberate forcing of the PPG unit had comparably poor PPG collection ability at the sternum (Figure S4, Supporting Information). Therefore, the passive PPG forcing mechanism was designed to force the PPG against the underlying skin via an elastomer block serving as a tunable spring. To hold the block in place during use, a laser‐cut, flexible mylar bracket was also adhered to the circuit. Naturally, larger blocks can generate more force, but increasing the elastomer height (and therefore sensor displacement) also prevents more of the surrounding adhesive area from remaining in contact with the skin, limiting the total force ultimately imparted on the PPG in a steady state. This trade‐off required empirical optimization to find the block thickness generating the maximum force with minimal variance over replicates of the experiment. Achievable PPG force was found for 0‐, 2‐, 4‐, and 6‐mm blocks using the experimental setup shown in Figure S5 and Video S1 (Supporting Information). Force was found to increase with elastomer height with approximately the same variance up to and including 4 mm, plateauing in average force with a larger variance at 6 mm. All group distributions were found to be significantly different from each other (*p* < 0.001) as indicated by the letter display in Figure 2i. Seeking to maximize contact pressure consistently, the 4 mm elastomer block height was selected for PPG forcing given the higher variance of the 6 mm group and negligible difference in the median generated force of the two groups. The PPG sensor was also placed on a novel flexible printed circuit board (fPCB) “island” with serpentine connections such that large out‐of‐plane strains of the sensor from the passive forcing remained possible with no broader deformation of the fPCB or excessive local strain. FEA of the island's interconnect design in Figure 2j confirmed that even with an excessively high force for the PPG forcing mechanism of 1N, local interconnect strain peaks at 2.0%. Note that all candidate elastomer block heights shown in Figure 2i reported achievable PPG force values less than what was simulated in Figure 2j, indicating the suitability of the interconnect design for the considered elastomer blocks.

### 2.3 Study of a Device's Multi‐Sensing Performance

**Figure 3:**
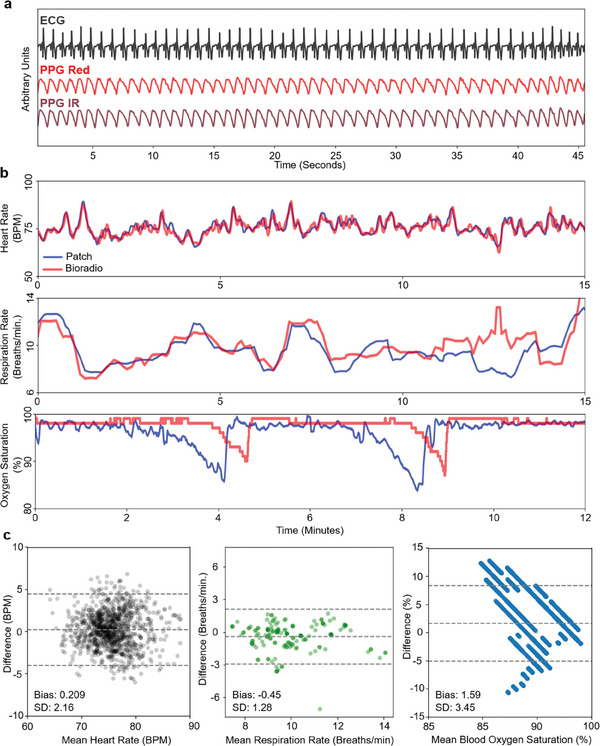
Soft device sensing characterization. a) Synchronized key cardiovascular waveforms, highlighting the achievable signal quality. Comparisons with the gold standard for b) from top to bottom, heart rate, respiration rate, and blood oxygen saturation, broadly showing agreement with the reference device. Blood oxygen saturation exhibits a time delay between finger and chest PPG sensors. c) Heart rate, respiration rate, and blood oxygen saturation Bland‐Altman plots are shown respectively for b).

Our wearable patch's sensing ability was characterized by comparison with a clinical‐grade reference device (BioRadio) with conventional gel electrode ECG and finger PPG sensing. **Figure** **3**a presents representative and aligned ECG and PPG waveforms from this controlled setting. Both the amplitude modulation of the ECG R‐peaks from which the respiration rate is derived and the dichroitic notch of the PPG are visible. Figure 3b overlays derived metrics of heart rate, respiration rate, and blood oxygen. Heart rate variability, while calculated by the cloud platform for its importance in arrhythmia detection among other use cases, was not provided by the reference device. These measurements, central to cardiovascular health characterization and presented to both patients and clinicians, show broad agreement with the reference measurements. This is confirmed by Bland–Altman plots for the same period shown in Figure 3c. There is a notable lag in the SpO_2_ reported by the reference device compared to the postpartum monitor. To determine whether this was a result of the distance from the pulmonary system to both the finger and the sternum or simply an artifact in the SpO_2_ reporting from the reference device, a follow‐up study was conducted as described in the methods to compare re‐saturation timing between sensors at the finger, chest, and toe, the results of which are shown in Figure S6 (Supporting Information). Using the return to saturation as a shared landmark, chest PPG saw a ≈30 s detection lead compared to finger sensors. The reference device and an identical PPG sensor (MAX30102) on the chest showed exact response times at the finger, reinforcing this finding and the previously observed lag in the reference sensor. Continuing this trend, the toe sensor was ≈30 s delayed. While there is some disruption likely due to subject motion during this measurement, the re‐saturation point is pronounced. It makes for nearly a minute difference between the chest and toe in terms of saturation detection. This has important implications for wearable systems designed for real‐time monitoring, as this example illustrates the role of sensing location (and the sternum's optimality thereof) in a wearable device's ability to monitor the patient's physiological state with respect to oxygen saturation.

### 2.4 Validation of a Device's Clinical Feasibility

**Figure 4:**
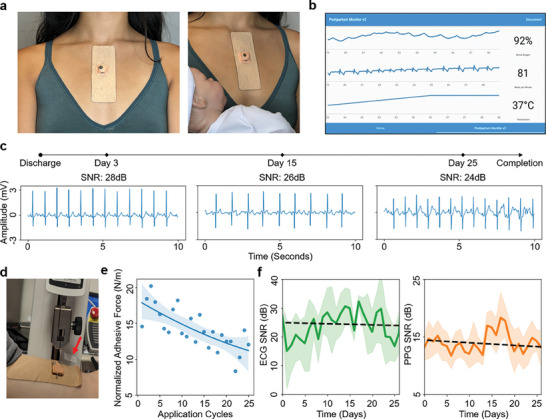
Device use case and sensing ability over month‐long, at‐home study. a) Demonstration of device usage in a postpartum application. b) Monitoring view for the subject who uses the wearable device. c) Study timeline and an example of ECG quality on select days. d) Mechanical testing setup with connection to device indicated. e) Testing of adhesive strength over a month's worth of device applications with quadratic best fit, indicating more than 50% of device adhesion remains after a month of monitoring. f) ECG (left) and PPG (right) mean SNR observed each day of the study, with 95% confidence intervals and linear fits shown.

This system, including wearable patch, mobile device and application, and cloud platform, was validated in an at‐home study with 20 Black women during their first month postpartum. A pair of devices was provided to each participant, classified by maternal health clinicians as belonging to a high or low‐risk population based on their medical history and health at discharge from the hospital. The high‐risk postpartum group was defined as those individuals with any history of cardiomyopathy, diabetes, thromboembolism, hypertension, or those who had ever given birth through Cesarean section. All of these are risk factors for postpartum complications defined in the study as the occurrence of infection, thromboembolism, hypertensive crisis, cardiomyopathy, heart failure, myocardial infarction, or hospitalization, the early manifestations of which this system was designed to monitor. The high‐risk experimental group contrasted with the low‐risk participants, who had no history of the high‐risk diseases and had no documented complications during their most recent pregnancy and subsequent childbirth, such as abnormal fetal development or Cesarean delivery. All participants were instructed to wear the device for a total of at least 10 min per day for the 28 days of the study to assess the system's ability to inform both the participants and the clinicians of the former's cardiovascular health (**Figure** **4**a). Participant ECG was used to calculate HR, RR, and HRV, while SpO2 and a second HR measurement were obtained from PPG (Figure 4b). Temperature readings were also considered by the clinic for interrogation of fever and infection. In addition to the two sensing devices, participants were provided a USB‐based magnetic charger (Figure S7, Supporting Information), an Android tablet loaded with the mobile application, and an informational video explaining the usage of the device. The tablet interface provided participants with real‐time feedback on heart rate, blood oxygen, and skin temperature and notified the user to adjust the fit of the device if poor data quality was detected by the cloud, as described previously in this work. These materials are documented in Figure 4a,b, which respectively show the patients' intended sensor placement and mobile device display. Video S2 and Figure S8 (Supporting Information) show typical device and mobile application use. A set of data in Figure 4c documents signal quality for a representative patient across the duration of the study. Though the retention of quality is not as high as the controlled testing, the quality is sufficient even on the final day of the survey for calculating HR, RR, and HRV. The ability of the device to undergo dozens of application cycles was explored to ensure its suitability for month‐long monitoring. Figure 4d and Video S3 (Supporting Information) display the mechanical testing used to quantify peel strength. After a month's worth of applications on clean skin, over 50% of adhesive strength remained, as seen in Figure 4e. Comparing this controlled study to the experimental mean ECG and PPG SNR plotted in Figure 4f, we suspect the curious long‐term rise and then fall observed for the ECG SNR to be a combination of initially increasing user proficiency in data collection followed by this adhesive degradation. For PPG, plotted on a narrower vertical scale than ECG, the abrupt shift within the third week of the trial corresponds to roughly +3 dB in mean SNR and may be spurious. Regardless, what signal quality remained after the full duration of the study was sufficient to retain over 95% of the original ECG and PPG signal quality on average. After the trial, over 40 h of submitted data were processed to assess the platform's ability to detect differences between the high‐risk and low‐risk populations. Data uploads were uniform throughout the study (Figure S9, Supporting Information).

**Figure 5:**
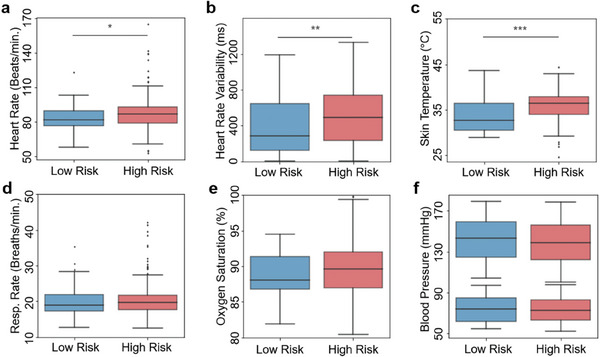
Remote clinical results obtained with the patch, separated by risk level. a) Heart rate, significantly elevated in the high‐risk group. b) Heart rate variability, also significantly (*p* < 0.01) elevated in the high‐risk group. c) Temperature, very significantly (*p* < 0.001) elevated in the high‐risk group. d) Respiration rate, with more outliers in the high‐risk population but otherwise showing similar distributions. e) Blood oxygen saturation, showing no difference in mean but a larger variance in the high‐risk group. f) Systolic (upper pair) and diastolic (lower pair) blood pressure distributions through a deep learning model, with no differences observed between groups for either.

**Figure** **5** summarizes remote clinical results obtained with wearable devices for differentiating risk levels. Statistically significant differences were observed between the two groups for HR (Figure 5a, *p* < 0.05), in line with known physiological manifestations of cardiovascular stress.^[^ ^19^ ^]^ Elevated HR (tachycardia) is especially predictive of cardiovascular for those with pre‐existing hypertension,^[^ ^20^ ^]^ which was the case for five of the 13 high‐risk participants and none of the low‐risk. The risk posed by tachycardia, as with the danger of postpartum complications, is known to scale with age and can manifest fatally as myocardial infarction and sudden cardiac death from ventricular fibrillation.^[^ ^21^ ^]^ Though still not entirely understood, the physiological origins of tachycardia and its implications on cardiovascular health are explored.^[^ ^20a^ ^]^ Thus, in detecting tachycardia, the device successfully identified an elevated risk for cardiovascular morbidity in the predicted experimental group. Separately, HRV, another known predictor of poor cardiovascular outcomes as detailed by Fink^[^ ^22^ ^]^ in a recent review, was also found to be higher in the high‐risk ground (Figure 5b, *p* < 0.01). Time domain measures such as the standard deviation of beat‐to‐beat intervals (reported in this work as HRV) were found to correlate negatively with higher risk. As reported in,^[^ ^22^ ^]^ reduced HRV is believed to represent impairments in the neural regulation of HR. HRV was also found to predict the onset of hypertension and myocardial ischemia. Conversely, however, the high‐risk group of this study showed elevated HRV. Given the sensitivity of the standard deviation to outliers, it is possible that the suboptimal fit of the device during the trial, in conjunction with the low sample size for each group of the study, skewed the beat‐to‐beat timing variances. Skin temperature was also significantly higher in the high‐risk group (Figure 5c, *p* < 0.001), though a relation between body temperature and cardiovascular risk is not well documented. No significant differences were observed between RR (Figure 5d), SpO_2_ (Figure 5e, *p* > 0.05), and BP (Figure 5f, *p* > 0.05) of high‐risk and low‐risk groups. Interestingly, systolic and diastolic pressures between both groups were not found to be different despite the hypertensive diagnoses of nearly half of the high‐risk population. Though perhaps surprising, 1st‐month postpartum individuals are under significant physiological and psychological stress as they recuperate from childbirth, which is known to create blood pressure confounders shared by both experimental groups.^[^ ^23^ ^]^

### 2.5 Demonstration of Calibration‐Free Blood Pressure Monitoring

As mentioned, hypertension is a key risk factor for postpartum complications. Standard noninvasive techniques in clinical practice for measuring BP rely on cuff‐based sphygmomanometers, which disallow continuous measurements and are generally uncomfortable. Conversely, recent wearable devices providing blood pressure predictions are shown in **Table** **2**, with many achieving this through pulse transit time (PTT) regression,^[^ ^24^ ^]^ which relates the propagation speed of the PPG pressure wave to arterial pressure. Unfortunately, PTT methods still require calibration via an invasive arterial line or a time‐consuming and less accurate blood pressure cuff. Neither the clinical nor wearable device PTT status quo is ideal for a wearable postpartum monitoring system, which should predict BP continuously and without requiring patients – many of whom already struggle to access clinical resources – from participating in potentially invasive calibration studies. Instead, we leveraged the public availability of large waveform databases for training a deep learning model. Specifically, we used the Multi‐parameter Intelligent Monitoring for Intensive Care (MIMIC) waveform database, which provides synchronized PPG, ECG, BP, and other waveforms from 10 000 intensive care unit patients. We selected 82 subjects with synchronized ABP and BP from the first iteration MIMIC‐I subset of the database, by prior PPG‐ABP machine learning work. This totaled 200 patient days of PPG and BP signals, which were processed with peak detection, bandpass filtering, and segmentation according to Figure S10 (Supporting Information). To extract as much information as possible from the PPG signal, the continuous wavelet transform (CWT) was used to generate spectro‐temporal data as images of 10‐s segments. The chosen model architecture, a deep residual convolutional neural network (CNN), reflects this input representation. CNNs are unique for their use of learned convolutional kernels, making them ideal for image processing. The large size of the input images necessitated many convolutional layers with down‐sampling to extract all useful information and to reduce the dimensionality of the feature set for the prediction of blood pressure with a final fully connected neural network. However, it has been known for decades that deeper networks present significant differences to learning due to vanishing or exploding gradients, wherein the networks cannot be trained further because the backpropagating signals used to adjust network weights are either very large or almost zero. Normalization of layer inputs in a process known as batch normalization (abbreviated batch norm) is known to mitigate these issues.^[^ ^25^ ^]^ However, deep networks leveraging this approach were still observed to lose accuracy with increasing depth, a problem addressed by^[^ ^26^ ^]^ in their seminal work on residual connections. For a given layer, residual or “shortcut” connections bypass its transformations and add the layer's unchanged input to its transformed output, preserving the features obtained up to that point by, in the extreme case, setting the layer's weights to zero and retaining only the original input. Preserving the identity mapping with each residual connection ensures the network will not both need to retain its learning and advance in the learning task within a single network block. This insight was an important development in the design of deep networks and remains a fixture of the field almost a decade later. As is standard for deep residual networks, we developed a repeated block structure in which successive blocks lower their output resolution but increase the number of output image channels (so‐called “feature maps”). This implementation detail is critical to the model's performance, as downsampling increases the receptive field – the region in the input image a filter in a deeper layer can learn from – and provides some invariance to transformations of the original image. These outcomes are of course desirable for real‐world scalogram data, where our spectral content of interest may shift within a segment or have a meaningful interaction with content elsewhere in that segment.

**Table 2:** Recent blood pressure prediction models integrated in wearable systems.

Reference	Model	Subjects	Meeting the AAMI and FDA standards	
			SBP	DBP
This work	Residual CNN	82	✓	✓
[24a]	Linear regression	21	−	−
[24b]	Linear regression	44	−	✓
[24c]	Linear regression	23	−	✓
[24d]	Linear regression	3	✓	✓
[24e]	Linear regression	2	−	−
[37]	ANN	35	−	−
[38]	CNN	4	✓	✓

AAMI, Association for the Advancement of Medical Instrumentation; FDA, U.S. Food and Drug Administration

**Figure 6:**
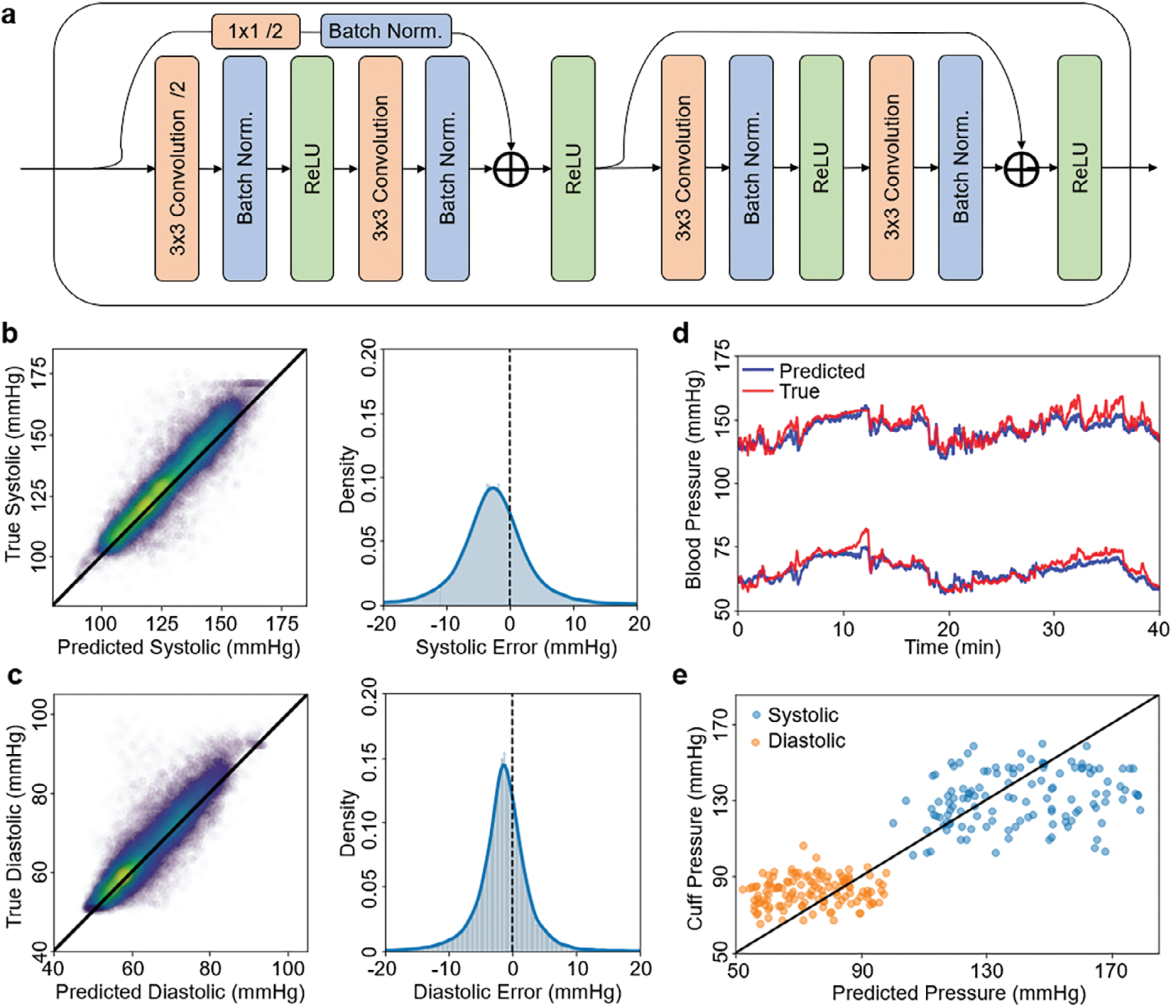
Architecture and performance of deep learning‐based blood pressure prediction from PPG. a) Residual‐convolutional block which doubles the feature map output while downsampling the input. b) Systolic and c) diastolic pressure predictions on the test dataset. Parity plots (left) and error distributions (right) indicate most predictions are within ± 10 mmHg of the true value. d) Prediction performance on the test (i.e., unseen during training) segment of arterial blood pressure data for systolic pressure (top) and diastolic pressure (bottom). e) Comparison of patient cuff measurements versus same‐day, wearable patch‐derived BP; there is a general agreement, and this discrepancy may come from intra‐day differences in timing between the patch and cuff measurements.

To achieve this, the modular network block of **Figure** **6**a was developed based on standard residual CNN design to both downsample the incoming feature maps from the previous block and perform its own feature extraction for downstream blocks. The network begins with an initial large‐kernel convolution, followed by three such blocks designed for 64‐, 128‐, and 256‐channel outputs respectively, with each output therefore downsampled by a factor of two. Within each block, we use batch norm and rectified linear unit activations, along with stridden convolutions for downsampling. The full architecture is provided in Figure S11 (Supporting Information). Training on the image stack provided from transforming and labeling the MIMIC data subset, we achieved 4.84 ± 4.16 mmHg systolic (Figure 6b) and 2.86 ± 2.97 mmHg diastolic (Figure 6c) mean absolute error and standard deviation on the held‐out test data, meeting the Association for the Advancement of Medical Instrumentation and U.S. Food and Drug Administration standards for clinic‐grade blood pressure prediction (5 ± 8 mmHg). The results in Figure 6d capture this performance, with predicted and true systolic and diastolic pressures closely agreeing for over 40 continuous minutes of test data.

Prior deep learning work has achieved lower error with stateful neural networks,^[^ ^27^ ^]^ but these were performed with smaller population sizes than our training set of 87 patients. With this number of patients from the MIMIC database, our dataset represents one of the largest subject counts in the literature for learning ABP from PPG in a wearable device system. Using the network and its preprocessing to the PPG received during the clinical trial for comparison with results from a blood pressure cuff provided to the patients by the clinic (Figure 6e), we see general agreement. This discrepancy is intra‐day differences in timing between the patch and cuff measurements and potentially more fundamental difficulties in predicting blood pressure at the sternum using models trained at the finger. Nonetheless, as summarized in Table 1, this represents one of the first efforts in leveraging contemporary cloud and machine learning developments to serve continuous blood pressure prediction in real‐time to at‐risk patient groups.

## 3 Conclusion

Collectively, this paper reports the development of a cloud‐integrated smart nanomembrane wearable system for remote, wireless, and continuous health monitoring of postpartum Black women, an underserved patient group in the United States. Our key contribution, validated in a pilot study, is the marriage of a smart sensor, consumer mobile devices, cloud‐based analytics, and calibration‐free blood pressure for long‐term physiological monitoring in low‐resource environments. Deep learning techniques on spectro‐temporal PPG features are a valid alternative to classical PTT approaches for real‐time, accurate blood pressure prediction. Indeed, the required recalibration procedures of PTT techniques render them unsuitable for patient populations lacking reliable access to medical infrastructure. Thus, we encourage future work to continue the pursuit of calibration‐free techniques for ambulatory blood pressure prediction in underserved populations. These technologies improve postpartum and broader NCD healthcare delivery by using integrated sensors and electronics in a telemedicine context to broaden screening, increase patient awareness, and improve clinical accessibility – directly addressing deficits in current clinical practice for these disease groups as evidenced by its ability to detect meaningful differences between the high‐ and low‐risk populations in the presented at‐home study. In future work, we will continue investigating effective telemedicine for patient groups in low‐resource environments. This portable and wearable platform could support additional sensors for detecting glucose, activity, and postoperative signals. Further, cloud integration presents opportunities for novel algorithmic development in the wearable device space, for example, by expanding on the broad portfolio of modern deep learning techniques such as transformers to include waveform analysis. As “edge” applications (i.e., those with machine learning models hosted directly on a device) continue to be explored by industry, cloud computation could be replaced in successor works entirely by highly optimized, fine‐tuned local models.

## 4 Experimental Section

### Nanomembrane Electrodes

Electrode substrate preparation consisting of 15.2 µm polyimide film on polydimethylsiloxane (PDMS)‐coated glass base followed the protocol discussed in prior work.^[^ ^13c^ ^]^ Electron beam deposition was used to evaporate the electrode materials, plating the substrate with high spatial resolution. A 200 nm copper base layer was used to minimize the electrical impedance of the electrodes. A 10 nm chromium adhesion layer followed by a 100 nm gold layer finalized the deposition process, providing a skin‐safe, low‐resistance, mechanically robust nanomembrane composite. The electrode pattern was excised from the plated slides using a 1030 nm femtosecond laser, the ultra‐short pulse duration of which permitted the creation of a compact electrode geometry by minimizing the imparted thermal loads on the target.

### PPG Forcing

To optimize the height of the elastomer block, a force‐sensitive resistor (FSR) (UNEO, GD‐03B) was inserted between the sensor and chest, and the force between the PPG sensor and the chest was measured for different elastomer thicknesses. Forcing was measured for blocks with 0‐, 2‐, 4‐, and 6‐mm heights. Calibration of the FSR was performed using a Mark‐10 ESM303 1.5 kN motorized test stand with a Mark‐10 M5‐5 25 N force meter and BK891 LCR meter. The calibration procedure involved cycling a sternum analog into the PPG sensor and measuring the relationship between force and resistance. The sternum analog was manufactured by sewing a piece of synthetic skin (SynDaver, Adult Skin 2N) to a plastic rigid backing.

### Sensing Electronics

A custom fPCB was designed to use an nRF52 microcontroller at a 3.3 V logic level. Interfaced with this via the SPI protocol is a 24‐bit ADS1292 analog‐to‐digital converter (ADC) sampling at 250 Hz for ECG collection. The nanomembrane electrodes interfaced with the fPCB and, thus, the ADC using anisotropic conductive film (ACF). The PPG was collected via the 18‐bit, two‐channel MAX30102 sampling red and infrared absorbance at 50 Hz, interfaced with the microcontroller via I2C. Sharing this I2C bus was a TMP117 12‐bit temperature sensor sampling at 1 Hz. The device used a 120 mAh rechargeable battery, which provided ≈24 h of continuous monitoring ability at a steady‐state current draw of 4–5 mA.

### Finite Element Analysis

Both the electrode pad and interconnect geometries were investigated under load using FEA in Ansys Mechanical (Ansys, Inc.). Given that nearly all the electrode cross section belonged to the polyimide substrate, the electrodes were modeled uniformly as 0.01 mm‐thick polyimide with isotropic properties, namely elastic modulus E = 2.4 GPa, Poisson ratio ν = 0.4, and density *ρ* = 1380kg m^−3^. The fPCB was also investigated using the same material properties assuming a uniform thickness of 0.1 mm.

### PPG Follow‐Up

A postpartum monitor at the sternum and the reference device at the finger were prepared for simultaneous measurements to replicate the lag in saturation observed in Figure 3b. In addition to these, another MAX30102 PPG sensor was placed against a finger on the same hand as the reference device and a toe on the same side of the body as both finger sensors. All MAX30102 devices were connected simultaneously, making use of the timestamp synchronization discussed below. The reference device was connected separately to a laptop, and the local system time for the mobile application was calibrated to the National Institute of Standards and Technology servers.

### Human Subject Study

Following a protocol approved by the University of Illinois Chicago (UIC) Institutional Review Board (#2020‐1162), 20 pregnant Black women were recruited in collaboration with the UIC Nursing School. After delivery, maternal health clinicians classified the participant as high or low‐risk based on the individual's medical history. Signed consent forms with written and verbal consent were received by the clinicians before discharge from the hospital in preparation for at‐home measurements, which included both the wearable system described here and a supplemental blood pressure cuff and finger blood oxygen sensor. Participants were provided an informational video on expected wearable device usage, which involved streaming data to the cloud for at least 10 min each day for their first 28 days after returning home.

### Statistical Methods

PPG forcing significance proceeded as follows: Levene's test confirmed that the groups exhibited significantly different variances (*p* < 0.01), and so popular analysis of variance (ANOVA) techniques for pairwise significance that assume homogeneous variance, such as Tukey's range test could not be employed soundly. Games–Howell testing, a nonparametric form of Tukey's range test with no assumptions of normality or variance homogeneity, was used instead under the null hypothesis that all group means are equal. Separately, Welch's *t*‐tests were used to assess differences between the high and low‐risk groups in the clinical data of Figure 5.

### Adhesive Strength

Adhesive strength over 28 application cycles was measured using a Mark‐10 force meter. Each cycle consisted of a peel test from cleaned forearm skin at a constant rate of 3 mm s^−1^ (Video S2, Supporting Information), followed by a measurement session with placement at the sternum for quantifying ECG and PPG SNR. The average force was found for each peel test and normalized by the width of the patch, while SNR was calculated in decibels as:

(1) $$\mathrm{SN}R_{\mathrm{db}}=20\;\;\log_{10}\left(\frac{x_{\mathrm{signal}}\left(t\right)}{x_{\mathrm{noise}}\left(t\right)}\right)$$

### Multiplatform Cloud Integration

A mobile application compatible with Android and iOS was developed using the Flutter development framework. Both major mobile operating systems can be targeted with Flutter using a single codebase, reducing development time and project complexity while expanding the scope of the wearable device platform to cover nearly all mobile devices currently in consumer use. Google Cloud Platform was chosen as the cloud provider for its compatibility with the Flutter SDK.

### Global Time Synchronization

Accurate timestamping of received data allows for direct comparison to data collected by other devices. Still, its implementation is complicated by a lack of obvious ground truth times available to wearable device systems. Mobile device (that is, tablet and smartphone) system time is a natural choice, but local time on consumer mobile devices can vary from true time on the order of seconds, an unacceptable error when comparing biosignal waveforms. Though the operating system of these devices periodically synchronizes itself with true time automatically and may provide the user with the ability to synchronize manually, it is impossible to derive the synchronization status of a mobile device from its uploaded data alone, making comparisons between data dubious without a deliberate synchronization solution. Thus, while still making use of the timekeeping convenience provided by system time, the existing internet connection was leverage necessary for cloud integration to synchronize the tablet with atomic clocks via the Network Time Protocol (NTP). At application startup and tunable intervals thereafter, the tablet corrects for any local system time deviations from true time by referencing these atomic clock‐backed servers. Synchronizing in this way places the device‐timestamped BLE packets on a global timeline shared by any other device with similarly accurate timestamping, such as conventional clinical monitoring equipment or other devices operating within this wearable system.

### MIMIC Dataset Preprocessing

PPG was bandpass filtered from 0.8 to 8 Hz with a fourth‐order Butterworth filter. Peak‐finding was then performed on the ABP and negative ABP signal for systolic and diastolic peaks respectively, and both the filtered PPG and its corresponding ABP were split into 10‐s segments without overlap. PPG segments with standard deviations in the interquartile range (Figure S12, Supporting Information) of each patient's distribution of segment standard deviation were selected; both high‐variance and low‐variance data were excluded because, as prior work has highlighted,^[^ ^29^ ^]^ real‐world biosignals such as these exhibit motion artifacts or periods of signal dropout that manifest as outliers in distributions of variance. Following segmentation and elimination of segments with extreme variance, scalograms were generated via Morlet CWT. In parallel with scalogram generation, peak‐finding was performed on the entirety of the patient's ABP waveform, and any segments with physiologically unreasonable systolic blood pressure (SBP) and diastolic blood pressure (DBP) such that 80 ≤ SBP ≤ 180 and 50 ≤ DBP ≤ 100 were ignored and the corresponding PPG segments dropped from the dataset. If all ABP peaks were found to be reasonable in a segment, its ABP peak amplitudes were averaged to produce a single pair of SBP/DBP labels for that segment (Figure S13, Supporting Information).

### Continuous Wavelet Transform

The continuous wavelet transform of a signal *x*(*t*) is defined as:

(2) $$X\;\left(\tau ,s\right)=\int_{-\infty }^{\infty }x\left(t\right)\frac{1}{\sqrt{\left|s\right|}}\psi \left(\frac{t-\tau }{s}\right)dt,\;\forall s,\tau \in R$$

for scales *s*, shifts τ, and where for a Morlet Wavelet, ψ(*t*)  = exp (*j*ω_ψ_ *t*) exp (− α*t* ^2^). The scales chosen of 20–140 for this wavelet approximate the passband of 0.8–8 Hz from the previously described MIMIC data bandpass filtering. The generated scalograms were 120 × 1200 images representing frequency content vertically and time horizontally, which were *z*‐score normalized individually (that is, normalized with respect to their own mean and variance). The distribution of scalogram values generated after transforming the input dataset is shown along with an example of PPG and ECG scalograms in Figure S14 (Supporting Information).

### Blood Pressure Machine Learning

With the data pipeline and model architecture shown respectively in Figures S10 and S11 (Supporting Information), training proceeded with a learning rate α of 0.001 using the Adam optimizer with momentum set to 0.9 and a batch size of 32. Early stopping at 20 epochs and a weight decay of 0.0005 were chosen to mitigate overfitting. Weight decay is a type of regularization in which the *L*_2_ norm of the network's weights is added to the network's function (analogously to ridge regularization in least‐squares regression). For a network with weights *w* and weight decay λ, the loss function L(x,w) for a batch of size *N* examples can be written as follows:

(3) $$L\;\;\;\;\left(x,w\right)=\frac{1}{N}\;\sum_{i\;=\;1}^{N}\frac{1}{2}{\left(w^{T}x_{i}+b-y_{i}\right)}^{2}+\frac{\lambda }{2}∥w{∥}_{2}^{2}$$

The network complexity itself thus becomes a point of optimization.

## Conflict of Interest

W.‐H.Y. and J.M. at Georgia Tech are inventors on a pending patent application related to this work.

## Supporting information

Supporting Information

Supplemental Video 1

Supplemental Video 2

Supplemental Video 3

## Data Availability

The data that support the findings of this study are available in the supplementary material of this article.
