# Supplementary material for: Cloud‐Integrated Smart Nanomembrane Wearables for Remote Wireless Continuous Health Monitoring of Postpartum Women
Source: Adv Sci (Weinh). 2024 Jan 26;11(13):2307609. doi: 10.1002/advs.202307609 (PMC10987106; doi:10.1002/advs.202307609)
Supplement: Supplementary file 1 — Supporting Information [file ADVS-11-2307609-s001.pdf]

ADVANCED SCIENCE
Open Access

# Supporting Information

for *Adv. Sci.*, DOI 10.1002/advs.202307609

Cloud-Integrated Smart Nanomembrane Wearables
for Remote Wireless Continuous Health Monitoring
of Postpartum Women

*Jared Matthews, Ira Soltis, Michelle Villegas-Downs, Tara A. Peters, Anne M. Fink, Jihoon Kim, Lauren Zhou, Lissette Romero, Barbara L. McFarlin and Woon-Hong Yeo**

## Supporting Information

**Cloud-Integrated Smart Nanomembrane Wearables for Remote Wireless Continuous Health Monitoring of Postpartum Women**

Jared Matthews[1,2], Ira Soltis[1,2], Michelle Villegas-Downs[3], Tara A. Peters[3], Anne M. Fink[4], Jihoon Kim[1,2], Lauren Zhou[1,2], Lissette Romero[1,2], Barbara L. McFarlin[3], Woon-Hong Yeo[1,2,5,6]*

[1]IEN Center for Wearable Intelligent Systems and Healthcare at the Institute for Electronics and Nanotechnology, Georgia Institute of Technology, Atlanta, GA 30332, USA.
[2]George W. Woodruff School of Mechanical Engineering, Georgia Institute of Technology, Atlanta, GA 30332, USA.
[3]Department of Human Development Nursing Science, College of Nursing, University of Illinois Chicago, 845 S. Damen Ave., MC 802, Chicago, IL 60612, USA.
[4]Department of Biobehavioral Nursing Science, College of Nursing, University of Illinois Chicago, 845 S. Damen Ave., MC 802, Chicago, IL 60612, USA.
[5]Wallace H. Coulter Department of Biomedical Engineering, Georgia Tech and Emory University School of Medicine, Atlanta, GA 30332, USA.
[6]Parker H. Petit Institute for Bioengineering and Biosciences, Institute for Materials, Neural Engineering Center, Institute for Robotics and Intelligent Machines, Georgia Institute of Technology, Atlanta, GA 30332, USA.
*Email: whyeo@gatech.edu

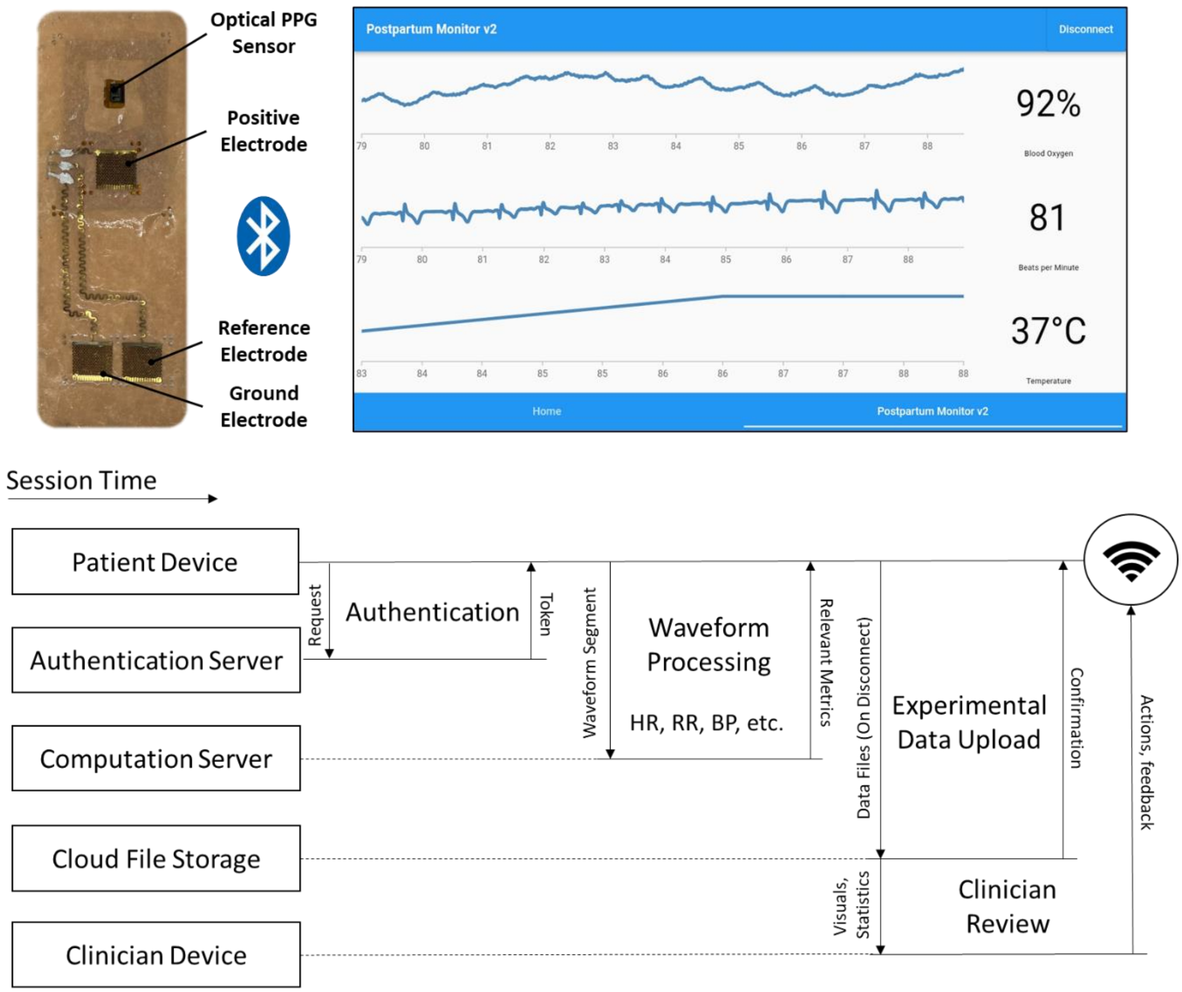


**Figure S1. Details of end-to-end system functionality**. The top image shows a system measuring data and the bottom shows the background cloud processes. All transitions are automated based on the state of the device; for example, waveform processing (beginning automatically on successful device connection) and bulk data upload (on device disconnect) require no user input.

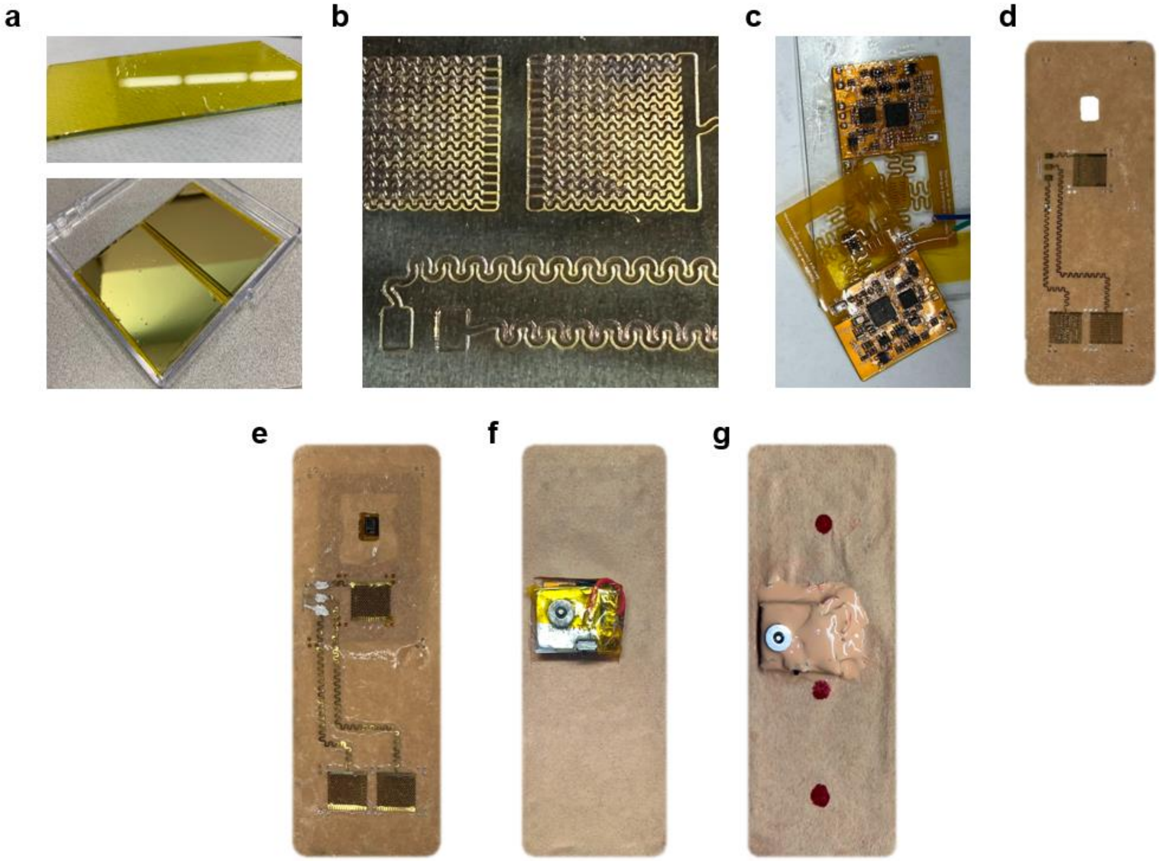

**Figure S2. Manufacturing process. a**, Polyimide film placement on glass slide spin-coated with polydimethylsiloxane (PDMS) (top) and result after deposition of copper, chromium, and gold layers via electron beam evaporation (bottom). **b**, Extraction of electrode pattern from plated slide using femtosecond pulse duration laser. **c**, Circuit manufacture and programming. **d**, Transfer of electrode pattern to patch substrate, medical tape with skin-safe Ecoflex adhesive coating cut to the desired profile. **e**, Installation of circuitry via epoxy and attachment of electrode to circuitry with anisotropic conducting film. **f**, Installation of battery and protective bilayer on reverse side **g**, Encapsulation of battery and circuitry with Ecoflex, with sensors marked for ease of patient use.

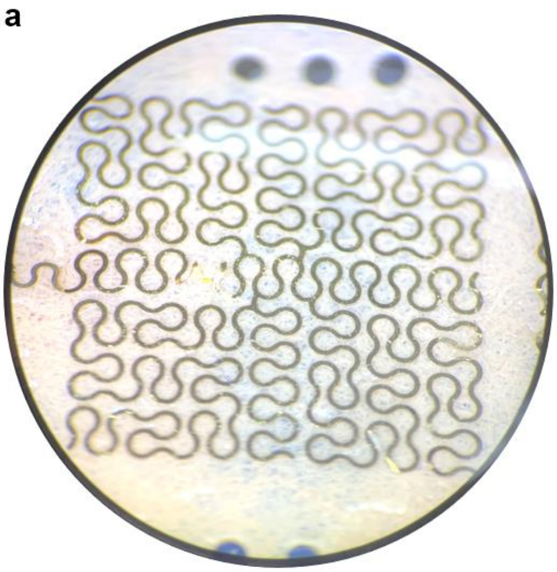


**Figure S3. Failure modes of classical electrode pattern after long-term use seen under light microscopy.** Degraded ECG can result from both (**a**) electrode delamination from adhesive and (**b**) delamination of gold film from PI substrate.

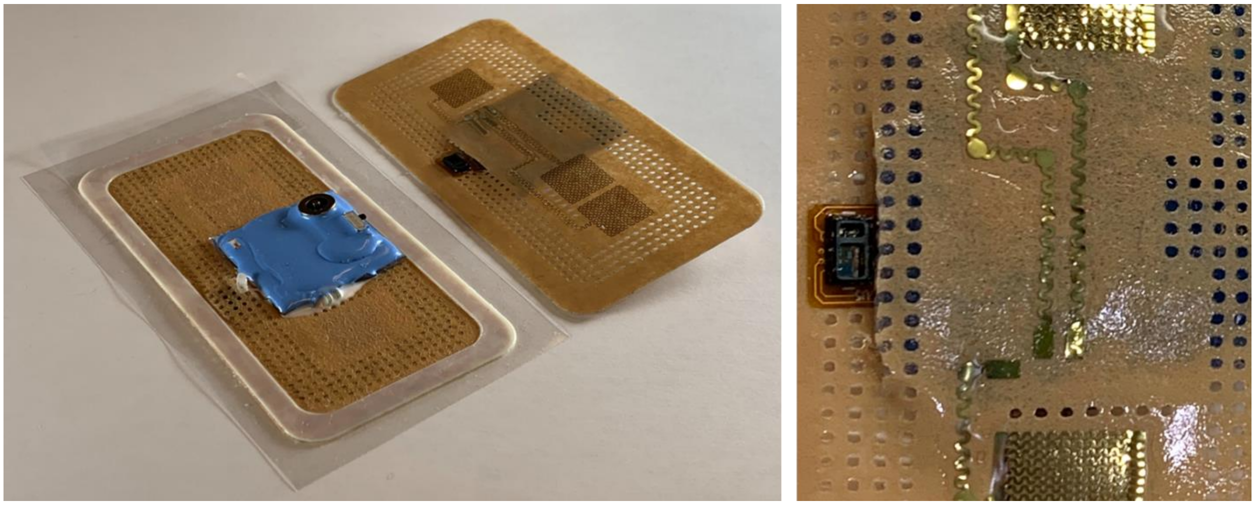

**Figure S4. Early device design with no PPG sensor forcing.** This device shows frustrated data collection at the sternum.

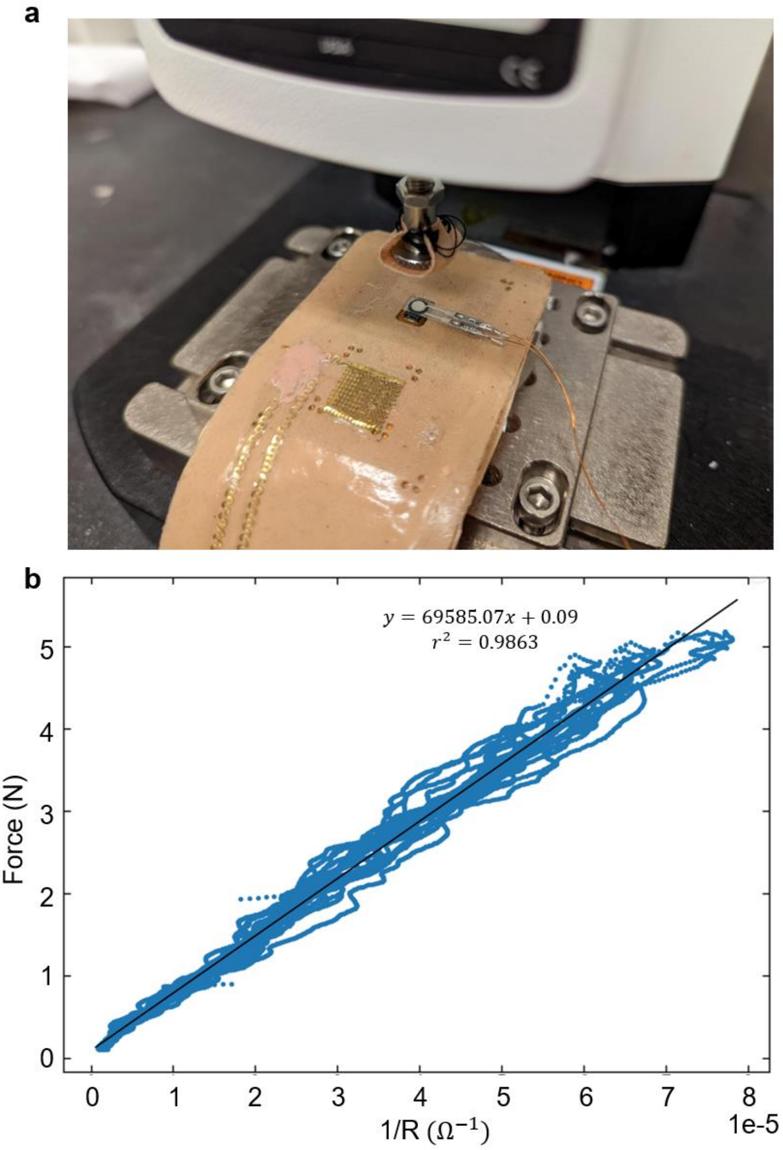


**Figure S5. PPG forcing calibration. a**, Experimental setup of force-sensitive resistor and **b**, obtained calibration curve (bottom).

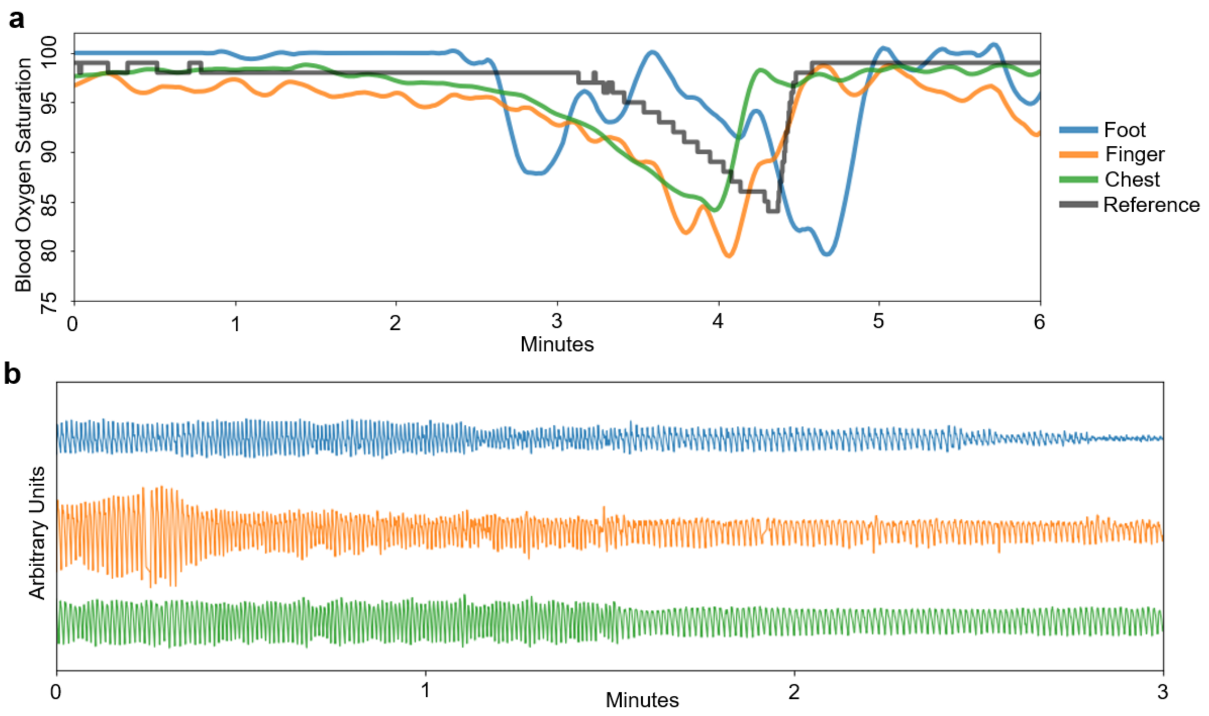


**Figure S6. Oxygen desaturation timing experiment. a**, Timing differences between chest, finger, and foot PPG sensors, with chest data providing the shortest response time. **b**, Corresponding PPG waveforms at onset of desaturation (same legend as top) show reduced amplitudes characteristic of hypoxic conditions at peripheral sites such as the toe.

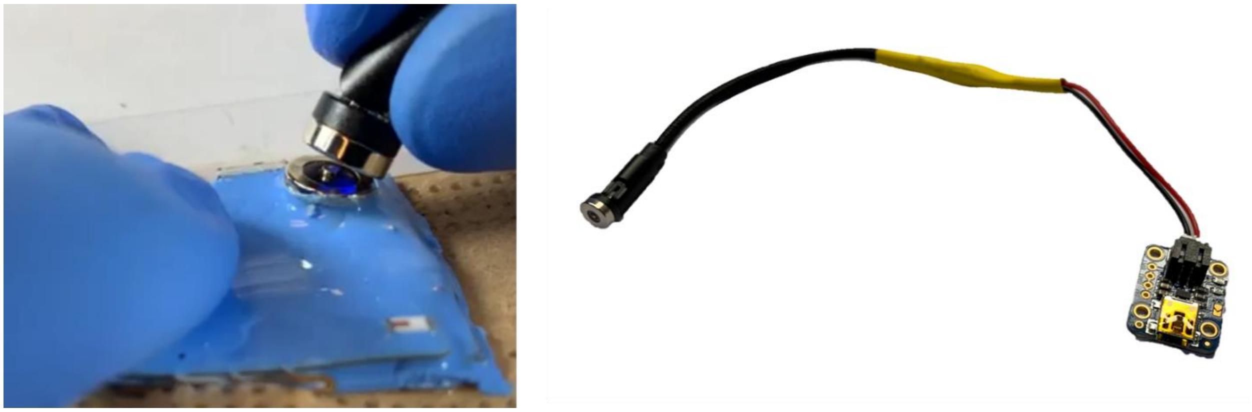

**Figure S7. Magnetic charging procedure (left) and flexible USB charging device (right).**

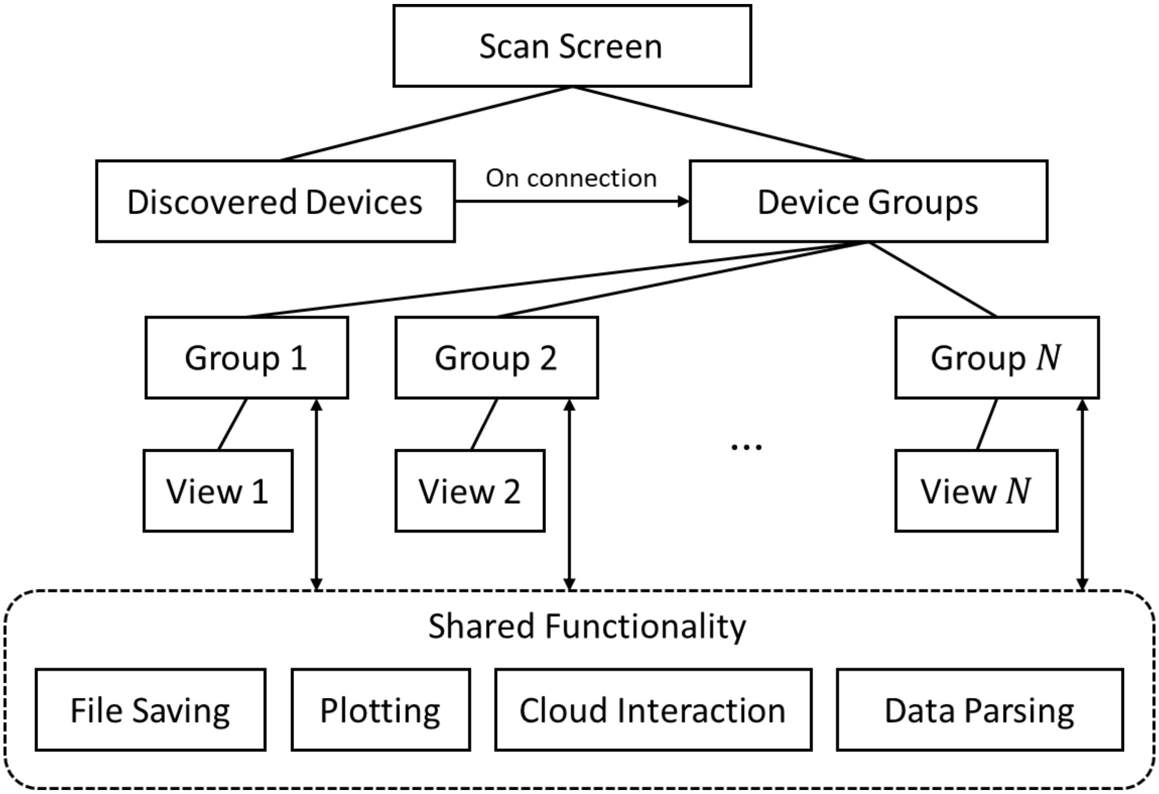


**Figure S8. Mobile application design.** Extensibility and modularity of the software demonstrated via arbitrary device grouping with shared core functionality.

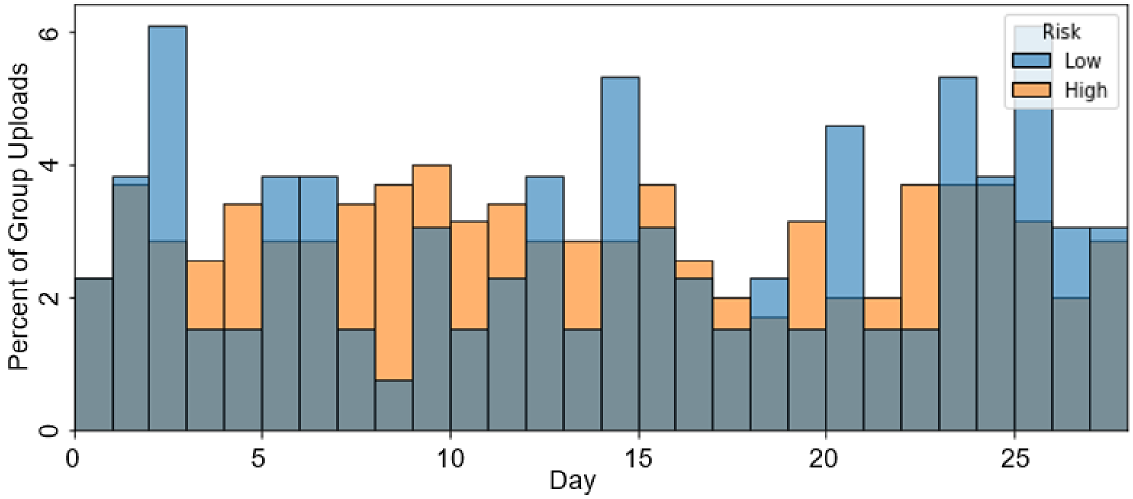


**Figure S9. Participant upload schedule.** Schedule of at-home study by risk stratification, indicating derived trends (e.g., heart rate, respiration rate) are informed by the entire month of the trial for both groups.

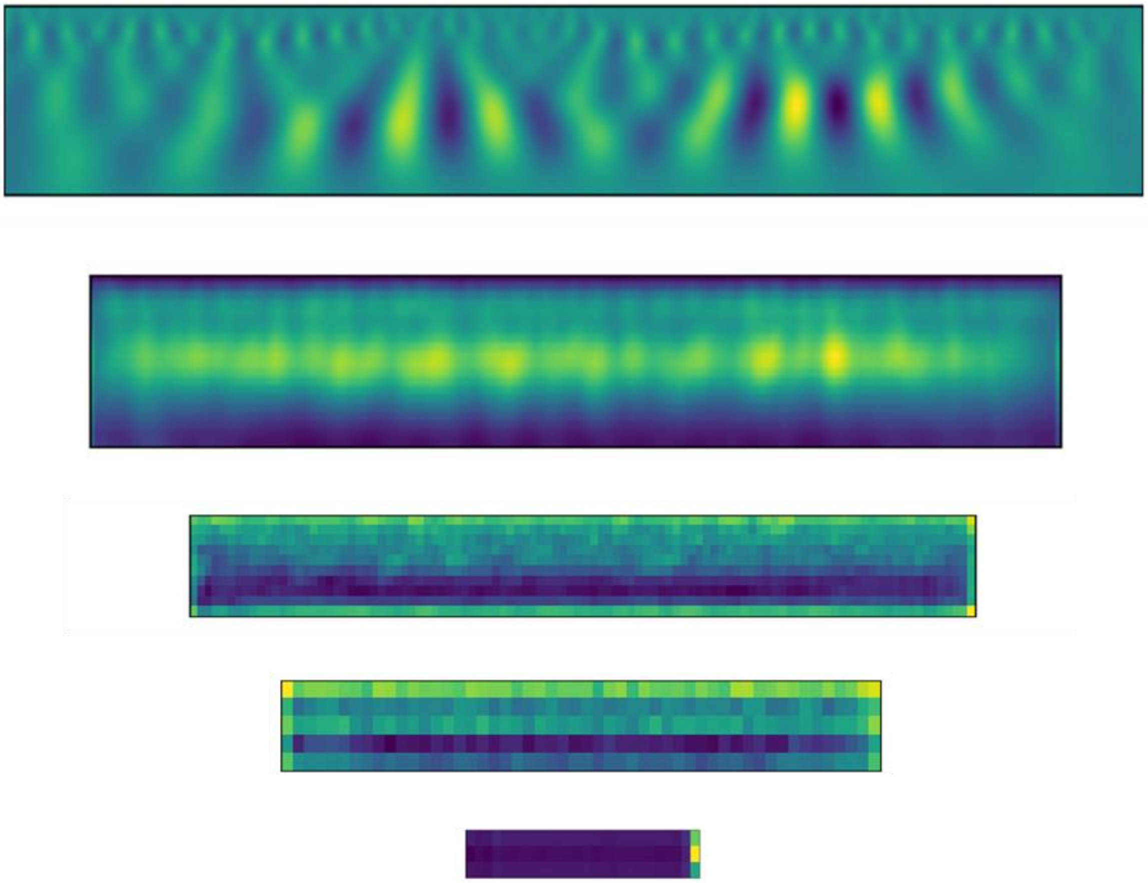

**Figure S10. Raw PPG scalogram input (top) and feature maps after successive residual blocks in the blood pressure neural network, averaged for an individual patient from the MIMIC dataset after model training.** The activations in the first residual block (second from top) may indicate the presence of learned representations derived from high-frequency features.

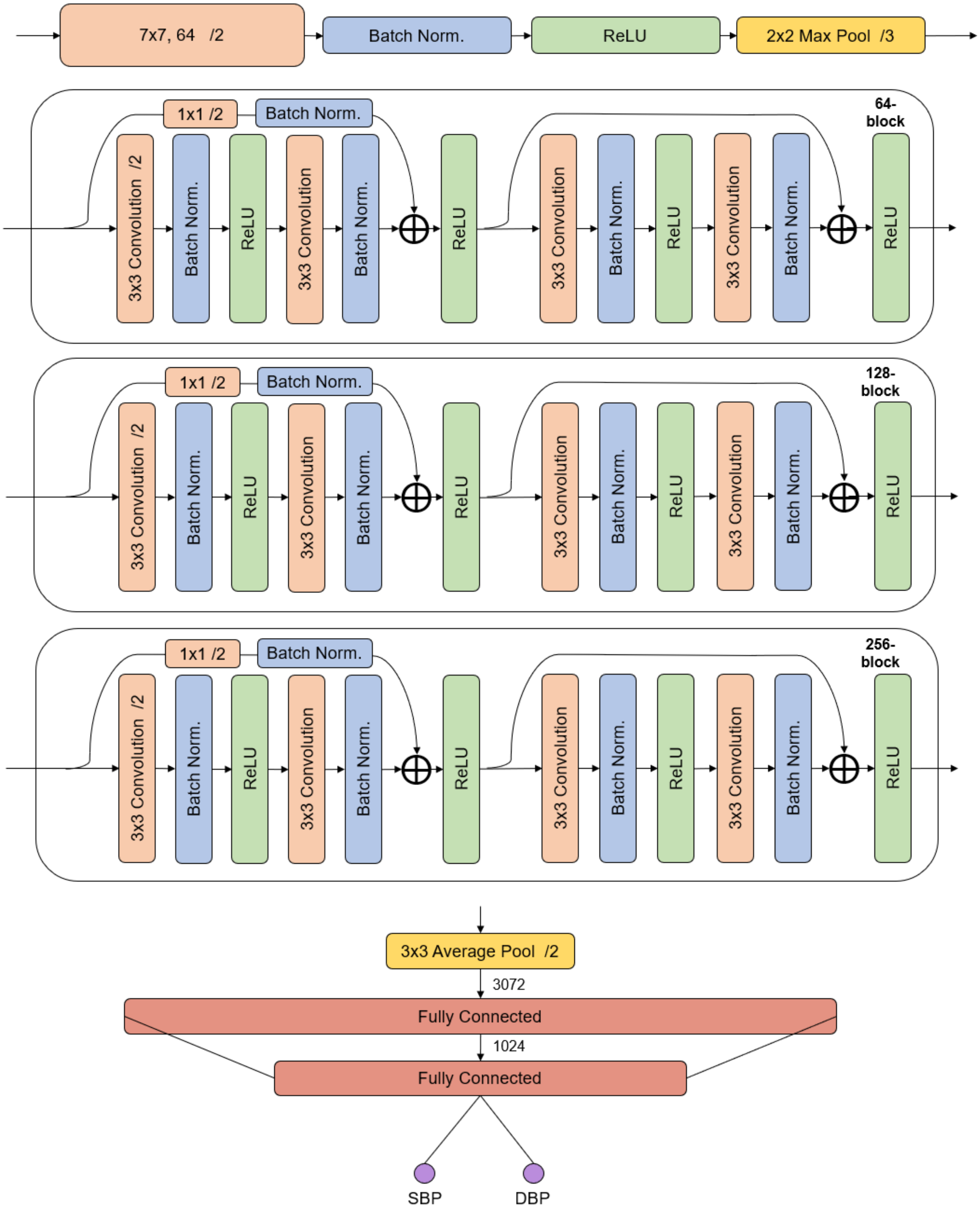


**Figure S11. Residual convolutional neural network architecture.** Network used for blood pressure prediction from PPG scalograms. A residual block based on the well-known ResNet architecture is repeated with increasing feature map count until the original image has been sufficiently downsampled for flattening as input into a feedforward network.

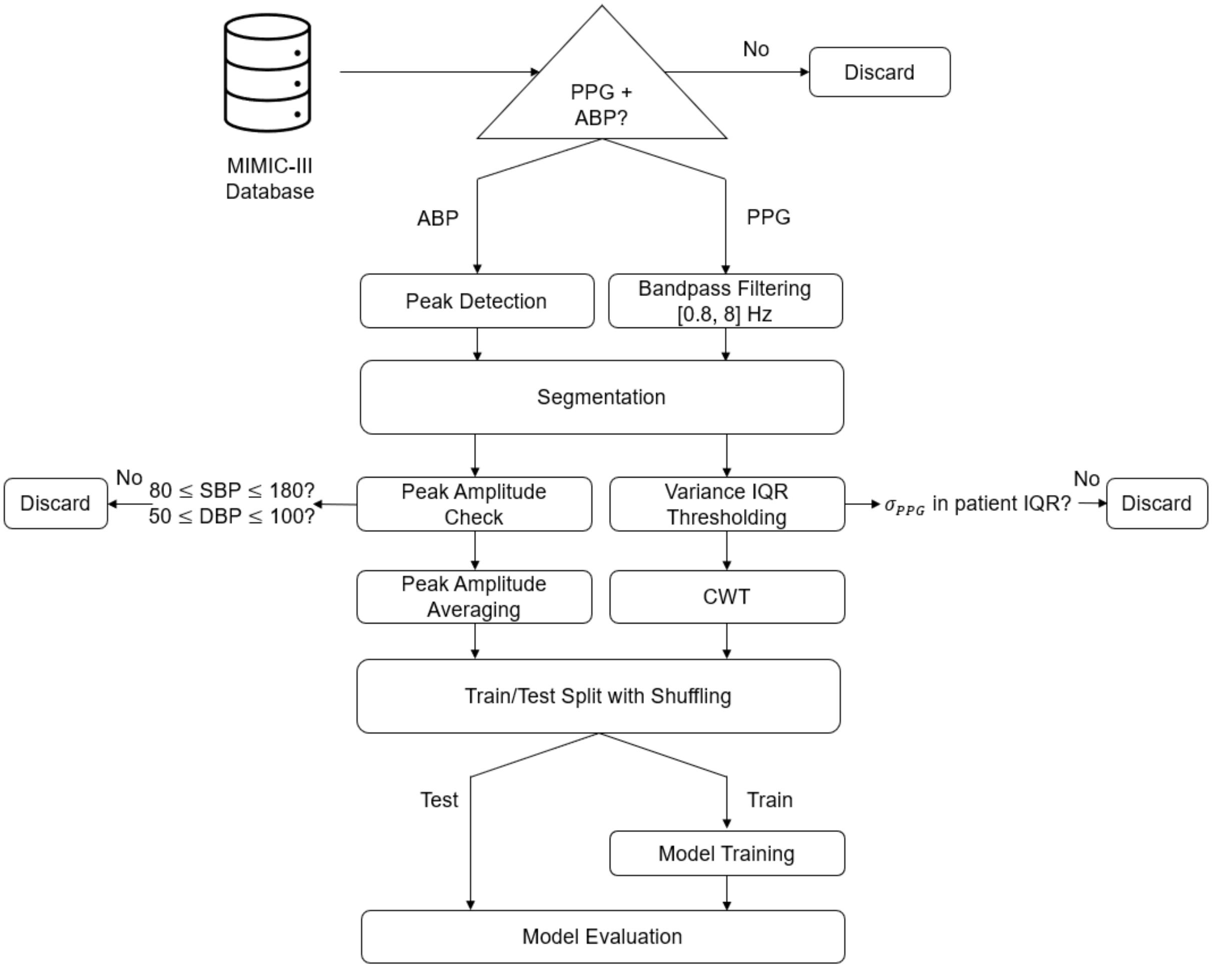


**Figure S12. Machine learning data pipeline.** Custom machine learning preprocessing, training, and evaluation pipeline showing interquartile range (IQR) thresholding and peak amplitude averaging.

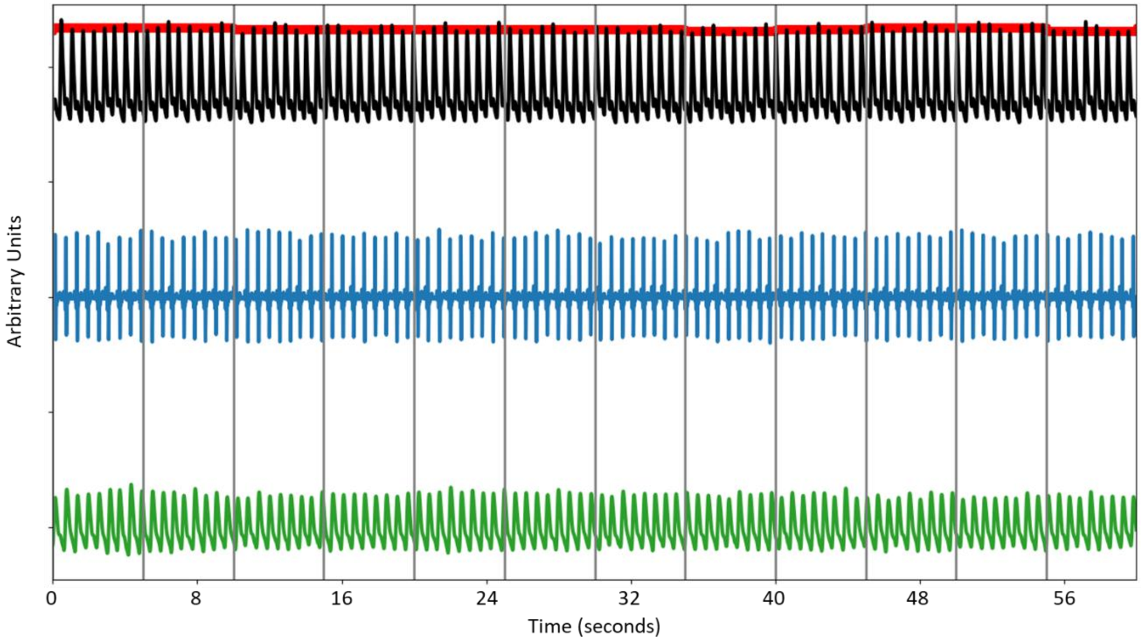


**Figure S13.** **Synchronized waveform processing for machine learning showing ABP (top), ECG (middle), and PPG (bottom). Segments are indicated by vertical bars.**

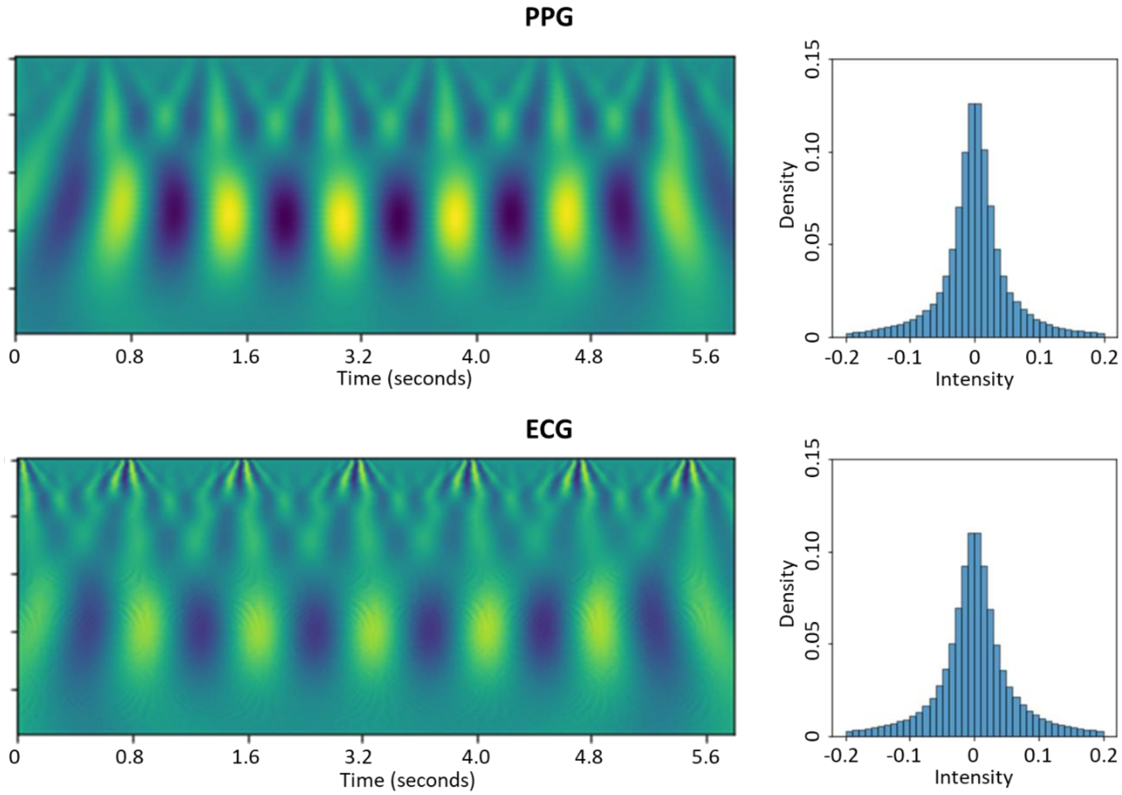


**Figure S14. Examples of PPG (top) and ECG (bottom) scalograms.** The multi-channel inputs to the machine learning model. Distributions of each channel's training data are shown to the right, illustrating the effect of normalization.

**Supporting Video 1** shows an experimental testing setup for PPG-forcing calibration.

**Supporting Video 2** captures an example of how the wearable system can wirelessly measure multiple cardiovascular signals using a cloud-integrated system.

**Supporting Video 3** shows an experimental setup for quantifying peel strength.
